# Uptake of hysterectomy and bilateral salpingo-oophorectomy in carriers of pathogenic mismatch repair variants: a Prospective Lynch Syndrome Database report

**DOI:** 10.1016/j.ejca.2021.02.022

**Published:** 2021-03-17

**Authors:** Toni T. Seppälä, Mev Dominguez-Valentin, Emma J. Crosbie, Christoph Engel, Stefan Aretz, Finlay Macrae, Ingrid Winship, Gabriel Capella, Huw Thomas, Eivind Hovig, Maartje Nielsen, Rolf H. Sijmons, Lucio Bertario, Bernardo Bonanni, Maria G. Tibiletti, Giulia M. Cavestro, Miriam Mints, Nathan Gluck, Lior Katz, Karl Heinimann, Carlos A. Vaccaro, Kate Green, Fiona Lalloo, James Hill, Wolff Schmiegel, Deepak Vangala, Claudia Perne, Hans-Georg Strauß, Johanna Tecklenburg, Elke Holinski-Feder, Verena Steinke-Lange, Jukka-Pekka Mecklin, John-Paul Plazzer, Marta Pineda, Matilde Navarro, Joan B. Vida, Revital Kariv, Guy Rosner, Tamara A. Piñero, Walter Pavicic, Pablo Kalfayan, Sanne W. ten Broeke, Mark A. Jenkins, Lone Sunde, Inge Bernstein, John Burn, Marc Greenblatt, Wouter H. de Vos tot Nederveen Cappel, Adriana Della Valle, Francisco Lopez-Koestner, Karin Alvarez, Reinhard Büttner, Heike Görgens, Monika Morak, Stefanie Holzapfel, Robert Hüneburg, Magnus von Knebel Doeberitz, Markus Loeffler, Silke Redler, Jürgen Weitz, Kirsi Pylvänäinen, Laura Renkonen-Sinisalo, Anna Lepistö, John L. Hopper, Aung K. Win, Noralane M. Lindor, Steven Gallinger, Loïc Le Marchand, Polly A. Newcomb, Jane C. Figueiredo, Stephen N. Thibodeau, Christina Therkildsen, Karin A.W. Wadt, Marian J.E. Mourits, Zohreh Ketabi, Oliver G. Denton, Einar A. Rødland, Hans Vasen, Florencia Neffa, Patricia Esperon, Douglas Tjandra, Gabriela Möslein, Erik Rokkones, Julian R. Sampson, D.G. Evans, Pål Møller

**Affiliations:** a Department of Gastrointestinal Surgery, Helsinki University Central Hospital, University of Helsinki, Helsinki, Finland; b Department of Surgical Oncology, Johns Hopkins Hospital, Baltimore, MD, USA; c Department of Tumor Biology, Institute of Cancer Research, The Norwegian Radium Hospital, Oslo, Norway; d Division of Cancer Sciences, Faculty of Biology, Medicine and Health, University of Manchester and St Mary’s Hospital, Manchester, UK; e Directorate of Gynaecology, Manchester University, NHS Foundation Trust, Manchester, M13 9WL, UK; f Institute for Medical Informatics, Statistics and Epidemiology, University of Leipzig, Leipzig, Germany; g Institute of Human Genetics, Medical Faculty, University of Bonn, Bonn, Germany; h National Center for Hereditary Tumor Syndromes, University Hospital Bonn, Germany; i Colorectal Medicine and Genetics, The Royal Melbourne Hospital, Melbourne, Australia; j Department of Medicine, Melbourne University, Melbourne, Australia; k Hereditary Cancer Program, Institut Catal. D’Oncologia-IDIBELL Institut D’Investigació Biomèdica de Bellvitge, L’Hospitalet de Llobregat, Barcelona, Spain; l St Mark’s Hospital, Department of Surgery and Cancer, Imperial College London, London, UK; m Department of Informatics, University of Oslo, Oslo, Norway; n Department of Clinical Genetics, Leids Universitair Medisch Centrum, Leiden, Netherlands; o Department of Genetics, University of Groningen, University Medical Center Groningen, Groningen, the Netherlands; p Division of Cancer Prevention and Genetics, IEO, European Institute of Oncology IRCCS, Milan, Italy; q Fondazione IRCCS Istituto Nazionale Dei Tumori, Milan, Italy; r Ospedale di Circolo ASST Settelaghi, Centro di Ricerca Tumori Eredo-familiari, Università Dell’Insubria, Varese, Italy; s Gastroenterology and Gastrointestinal Endoscopy Unit, Vita-Salute San Raffaele University, San Raffaele Scientific Institute, Milan, Italy; t Department of Women’s and Children’s Health, Division of Obstetrics and Gyneacology, Karolinska Institutet, Karolinska University Hospital, Solna, Stockholm, Sweden; u Tel-Aviv Sourasky Medical Center, Research Center for Digestive Disorders and Liver Diseases; v Department of Gastroenterology, Tel-Aviv Sourasky Medical Center and Sackler Faculty of Medicine, Tel-Aviv University, Israel; w High Risk and GI Cancer Prevention Clinic, Gatro-Oncology Unit, The Department of Gastroenterology, Sheba Medical Center, Israel; x Medical Genetics, Institute for Medical Genetics and Pathology, University Hospital Basel, Switzerland; y Hereditary Cancer Program (PROCANHE) Hospital Italiano de Buenos Aires, Buenos Aires, Argentina; z Instituto de Medicina Traslacional e Ingenieria Biomedica (IMTIB), Argentina; aa Manchester Centre for Genomic Medicine, Manchester University Hospitals NHS Foundation Trust, Manchester, UK; ab Department of Surgery, Manchester University Hospitals NHS Foundation Trust and University of Manchester, Manchester, UK; ac Department of Medicine, Knappschaftskrankenhaus, Ruhr-University Bochum, Bochum, Germany; ad Department of Gynaecology, University Clinics, Martin-Luther University, Halle-Wittenberg, Germany; ae Institute of Human Genetics, Hannover Medical School, Hannover, Germany; af Medizinische Klinik und Poliklinik IV, Campus Innenstadt, Klinikum der Universität München, Munich, Germany; ag MGZ- Medical Genetics Center, Munich, Germany; ah Faculty of Sport and Health Sciences, University of Jyväskylä, Jyväskylä, Finland & Department of Surgery, Central Finland Health Care District, Jyväskylä, Finland; ai The Royal Melbourne Hospital, Melbourne, Australia; aj Hereditary Cancer Program, Institut Català D’Oncologia-IDIBELL, L’Hospitalet de Llobregat, Barcelona, Spain; ak Centre for Epidemiology and Biostatistics, Melbourne School of Population and Global Health, The University of Melbourne, Parkville, Victoria, Australia; al Department of Clinical Genetics, Aalborg University Hospital, Aarhus, Denmark; am Department of Biomedicine, Aarhus University, Aarhus, Denmark; an Department of Surgical Gastroenterology, Aalborg University Hospital, Aalborg, Denmark; ao Faculty of Clinical Medicine, Aalborg University, Aalborg, Denmark; ap Faculty of Medical Sciences, Newcastle University, Newcastle Upon Tyne, UK; aq University of Vermont, Larner College of Medicine, Burlington, VT 05405, USA; ar Department of Gastroenterology and Hepatology, Isala Clinics, Zwolle, the Netherlands; as Grupo Colaborativo Uruguayo, Investigación de Afecciones Oncológicas Hereditarias (GCU), Hospital Fuerzas Armadas, Montevideo, Uruguay; at Lab. Oncología y Genética Molecular, Unidad de Coloproctología Clínica Las Condes, Santiago, Chile; au Institute of Pathology, University of Cologne, Cologne, Germany; av Department of Surgery, Technische Universität Dresden, Dresden, Germany; aw Institute of Human Genetics, University of Bonn, Bonn, Germany; ax Department of Internal Medicine I, University Hospital Bonn, Bonn, Germany; ay Department of Applied Tumour Biology, Institute of Pathology, University Hospital Heidelberg, Heidelberg, Germany; az Cooperation Unit Applied Tumour Biology, German Cancer Research Center (DKFZ), Heidelberg, Germany; ba Heinrich-Heine-University, Medical Faculty, Institute of Human Genetics, Düsseldorf, Germany; bb Department of Education and Science, Central Finland Health Care District, Jyväskylä, Finland; bc Department of Gastrointestinal Surgery, Helsinki University Central Hospital, Applied Tumour Genomics Research Program, University of Helsinki, Helsinki, Finland; bd University of Melbourne Centre for Cancer Research, Victorian Comprehensive Cancer Centre, Melbourne, VIC, 3010, Australia; be Genetic Medicine, Royal Melbourne Hospital, Parkville, VIC, 3050, Australia; bf Department of Health Science Research, Mayo Clinic Arizona; bg Lunenfeld Tanenbaum Research Institute, Mount Sinai Hospital, University of Toronto; bh University of Hawaii Cancer Center, Honolulu, HI 96813, USA; bi Public Health Sciences Division, Fred Hutchinson Cancer Research Center, Seattle, WA, 98109-1024, USA; bj Department of Laboratory Medicine and Pathology, Mayo Clinic, Rochester, MN, 55905, USA; bk The Danish HNPCC Register, Clinical Research Centre, Copenhagen University Hospital, Hvidovre, Denmark; bl Department of Clinical Genetics, Copenhagen University Hospital, Rigshospitalet, Denmark; bm Dept. of Obstetrics and Gynaecology, Copenhagen University Hospital, Rigshospitalet, Denmark; bn Institute of Medical Genetics, Division of Cancer and Genetics, Cardiff University School of Medicine, Cardiff, UK; bo Department of Gastroenterology and Hepatology, Leiden University Medical Centre, Leiden, the Netherlands; bp Department of Surgery, Ev. Krankenhaus Bethesda Hospital, Duisburg, Germany; bq Department of Gynaecological Oncology, Division of Cancer Medicine, The Norwegian Radium Hospital, Oslo, Norway; br Division of Evolution and Genomic Medicine, University of Manchester, Manchester, UK; bs Manchester Centre for Genomic Medicine, Manchester University NHS Foundation Trust, Manchester Academic Health Science Centre, Manchester, UK; bt The International Society for Gastrointestinal Hereditary Tumours (InSiGHT), The Polyposis Registry, St Mark’s Hospital, Watford Road, Harrow, Middlesex, HA1 3UJ, UK; bu European Hereditary Tumour Group (EHTG), C/o Lindsays, Caledonian Exchange, 19A Canning Street, Edinburgh, EH3 8HE, United Kingdom; bv Department of Gynaecologic Oncology, University of Groningen, University Medical Center Groningen, Groningen, the Netherlands

**Keywords:** Lynch syndrome, Endometrial cancer, Ovarian cancer, Risk-reducing surgery, Hysterectomy, Oophorectomy, *MLH1*, *MSH2*, *MSH6*, *PMS2*

## Abstract

**Purpose:**

This study aimed to report the uptake of hysterectomy and/or bilateral salpingo-oophorectomy (BSO) to prevent gynaecological cancers (risk-reducing surgery [RRS]) in carriers of pathogenic MMR (*path_MMR*) variants.

**Methods:**

The Prospective Lynch Syndrome Database (PLSD) was used to investigate RRS by a cross-sectional study in 2292 female *path_MMR* carriers aged 30–69 years.

**Results:**

Overall, 144, 79, and 517 carriers underwent risk-reducing hysterectomy, BSO, or both combined, respectively. Two-thirds of procedures before 50 years of age were combined hysterectomy and BSO, and 81% of all procedures included BSO. Risk-reducing hysterectomy was performed before age 50 years in 28%, 25%, 15%, and 9%, and BSO in 26%, 25%, 14% and 13% of *path_MLH1, path_MSH2, path_MSH6*, and *path_PMS2* carriers, respectively. Before 50 years of age, 107 of 188 (57%) BSO and 126 of 204 (62%) hysterectomies were performed in women without any prior cancer, and only 5% (20/392) were performed simultaneously with colorectal cancer (CRC) surgery.

**Conclusion:**

Uptake of RRS before 50 years of age was low, and RRS was rarely undertaken in association with surgical treatment of CRC. Uptake of RRS aligned poorly with gene- and age-associated risk estimates for endometrial or ovarian cancer that were published recently from PLSD and did not correspond well with current clinical guidelines. The reasons should be clarified. Decision-making on opting for or against RRS and its timing should be better aligned with predicted risk and mortality for endometrial and ovarian cancer in Lynch syndrome to improve outcomes.

## 1. Introduction

Lynch syndrome (LS) is a dominantly inherited cancer syndrome caused by germline pathogenic variants of mismatch repair (MMR) genes (*path_MMR* variants). In women with LS, gynaecological cancers are as common as gastrointestinal cancers.

No screening programme is considered to be effective for gynaecological cancers. Risk-reducing surgery (RRS), including total hysterectomy and bilateral sal pingo-oophorectomy (BSO), prevents gynaecological cancer in women with LS and is the only preventive approach that is recognised to be effective [1,2]. The Manchester International Consensus Group strongly recommended that risk-reducing hysterectomy and BSO is offered but no earlier than 35–40 years of age, following completion of childbearing in *path_MLH1, path_MSH2*, and *path_MSH6* carriers. There was insufficient evidence to strongly recommend RRS for *path_PMS2* carriers [3,4].

The distribution of ages at which RRS takes place in *path_MMR* women is not well known, and there is limited information on opportunistic RRS being undertaken in association with surgery for colorectal cancer (CRC). Undertaking RRS as the first major abdominal surgery before the occurrence of CRC constitutes a truly prophylactic procedure that may be performed on healthy *path_MMR* carriers. By contrast, some CRC patients are identified as *path_MMR* carriers after tumour MMR screening and are offered RRS as a secondary operation. In known *path_MMR* carriers, the timing of the RRS may avoid multiple surgeries if based on a predicted sequence of events with respect to CRC and the menopause. For women who choose not to undergo RRS, an understanding of ‘red flag’ symptoms (abnormal vaginal bleeding) is important to trigger prompt referral for urgent examination, and many centres provide gynaecological surveillance [5,6].

There is limited information on the uptake of RRS in *path_MMR* carriers, a corresponding lack of information on the extent to which clinical guidelines have been adopted and a lack of information on the alignment of gynaecological cancer risk and mortality with RRS uptake. In this report, we describe the uptake of hysterectomy and BSO reported to the Prospective Lynch Syndrome Database (PLSD) by age and gene and consider uptake in the context of recently published gynaecological cancer risk and mortality determined through PLSD.

## 2. Patients and methods

### 2.1. PLSD design

The PLSD is an international, multicentre, prospective observational study without a control group [7-10]. In brief, carriers of Class 4 or 5 pathogenic variants listed in the InSiGHT database (https://www.insight-group.org/variants/databases/), who had been recruited for prospective follow-up in each participating centre, are included. Inclusion was from the first prospectively planned and completed colonoscopy. The methods to define previous cancer, censoring of each patient, and observation time until organ removal have been previously described [7-10].

### 2.2. Ethics statement

All reporting centres exported deidentified data to the PLSD based on local institutional reviews, as previously described [7-10].

### 2.3. Selection criteria

The inclusion criteria for calculating the uptake of RRS were (1) female, (2) carrier of pathogenic or likely pathogenic (Class 4 or 5) MMR variant according to InSiGHT database classification [11], (3) aged 30–69 years at last examination, (4) no endometrial or ovarian cancer before or at inclusion age, and (5) at least 2 years of follow-up after first prospectively planned and carried-out colonoscopy (to ensure time from disclosure of carrier status to undertake RRS). The last observation was prospectively detected endometrial or ovarian cancer or last prospective examination without cancer.

In premenopausal women, hysterectomy may or may not be performed during treatment for early stage ovarian cancer, and BSO may or may not be performed during treatment of early stage endometrial cancer. Therefore, in all previous PLSD reports, when endometrial or ovarian cancer was diagnosed, observation time was right censored for the other organ. Correspondingly, in the present study, removal of the second organ during or after treatment for ovarian or endometrial cancer was not classified as an RRS procedure. RRS in this report indicates surgery for prophylaxis or for benign indications, unless otherwise specified.

### 2.4. Reported uptake of hysterectomy or BSO

In our analysis, we report total incidences of hysterectomy and BSO, and some of the interventions may not have been prophylactic surgeries *per se*, but organ removals for benign indications. Of note, BSO reported to the PLSD was specified as complete removal of both ovaries, which by current standards includes salpingectomy, reflecting the understanding that most high-grade serous ovarian cancers with serious prognosis may originate from the distal end of the salpinx [12]. We did not specifically ask about peritoneal cancer after BSO or endometrial cancer after hysterectomy [1].

### 2.5. Statistical methods

The following information was used for analyses: age at hysterectomy, age at BSO, age at last observation, and *path_MMR* variant.

The selected carriers were grouped in four 10-year cohorts categorised according to age at last observation. The numbers of carriers who had or did not have hysterectomy or BSO before or at last observation in each age cohort was counted, and the fractions of carriers who had these interventions in each category were calculated. The uptake of prophylactic surgery is reported as the cross-sectional frequency in each of the four different 10-year cohorts according to age at censoring.

In contrast to some former reports from the PLSD, this report is a cross-sectional study reporting age at last observation rather than annual incidences by age or cumulative incidences. The observation period was from birth to last observation because events that occurred before inclusion to prospective follow-up and reported by carriers were logged in PLSD and events after inclusion for follow-up were logged as reported by the collaborating centres.

## 3. Results

### 3.1. Inclusion of path_MMR carriers

Among the carriers included in the last PLSD version [10], 2292 female *path_MMR* carriers from 18 countries met the inclusion criteria for the current cross-sectional study (Supplementary Table 1). Of these, 1016, 833, 271, 152, and 20 were carriers of *path_MLH1*, *path_MSH2, path_MSH6, path_PMS2*, and *path_EPCAM*, respectively.

### 3.2. Uptake of risk-reducing hysterectomy and/or BSO

The mean ages at first RRS together with the mean ages at first CRC are presented by gene in Table 1. The mean age at first RRS was 45 years for *path_MLH1*, 44 years for *path_MSH2*, 48 years for *path_MSH6*, and 53 years for *path_PMS2* carriers, whereas the mean ages for first CRC were 41, 41, 44, and 47 years, respectively.

Of the 2292 *path_MMR* carriers aged 30–69 years, 664 (29%) had hysterectomy and 598 (26%) had BSO (Table 2). Of 1178 of 2292 carriers aged 30–49 years, 204 (17%) had hysterectomy and 188 (16%) had BSO (Table 2). At 40–49 years of age, the uptake for hysterectomy and/or BSO was 32% (102/320) and 30% (80/269) for *path_MLH1* and *path_MSH2*, respectively, whereas for *path_MSH6* carriers and *path_PMS2* carriers the uptake reached 18% (13/73) and 13% (4/32), respectively (Table 3).

As 144 (9.4%), 79 (3.5%), 517 (22.8%), and 1532 (67.4%) carriers underwent only risk-reducing hysterectomy, only BSO, both combined, or neither, respectively, 81% of surgical procedures included BSO (Table 3). Two-thirds (157/235, 67%) of procedures before age 50 years were combined hysterectomy and BSO.

**Fig. 1.**
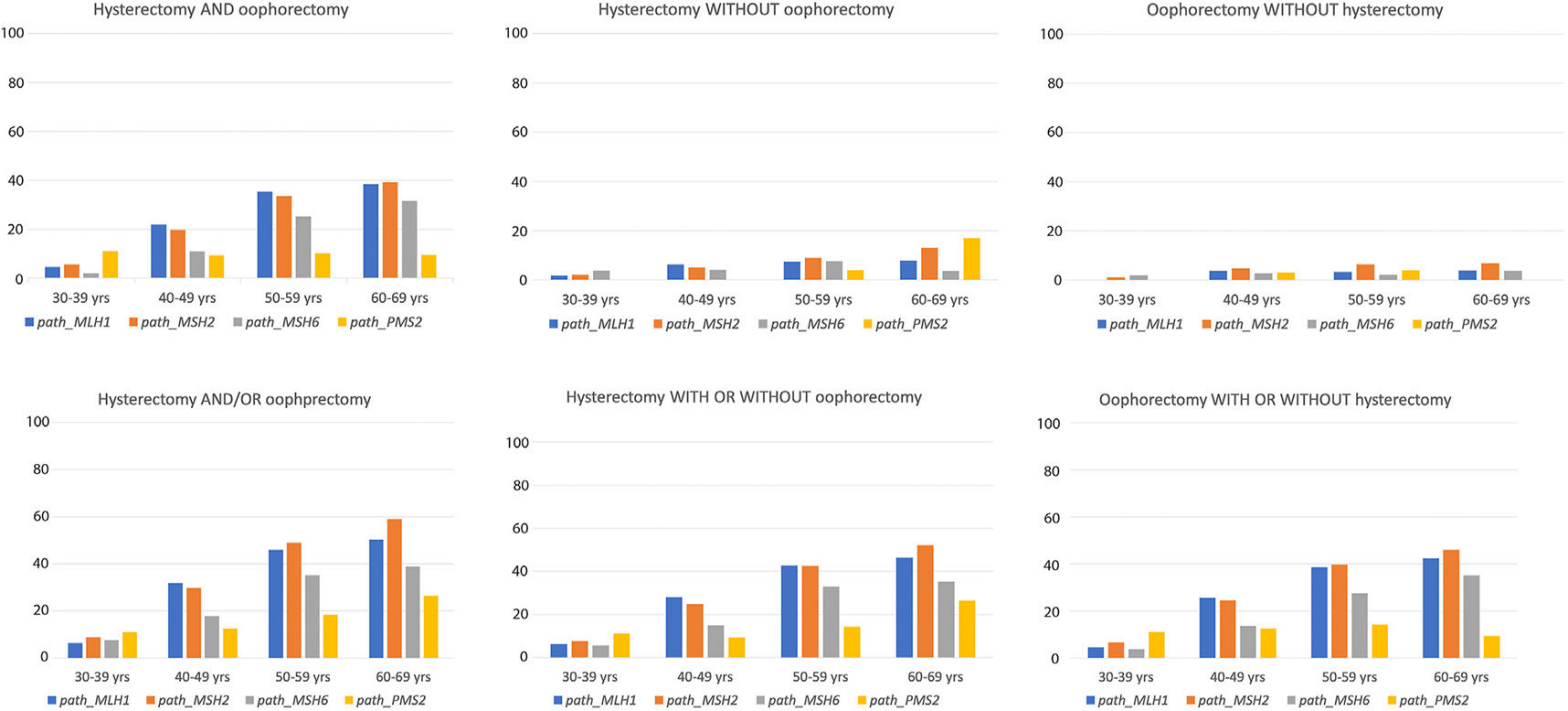
Uptake of hysterectomy and bilateral salpingo-oophorectomy (%) by age cohort and *path_MMR* gene.

The number of *path_EPCAM* carriers (N = 20) was too low for meaningful statistical analyses by gene and age, and they were excluded from the analysis (Table 3 and Fig. 1). Among the remaining 2272 *path_MMR* carriers, 342 *path_MLH1*, 299 *path_MSH2*, 70 *path_MSH6*, and 29 *path_PMS2* carriers had hysterectomy and/or BSO. The frequencies in the uptake of hysterectomies and BSO were calculated separately and in combination in 10-year age cohorts between 30 and 69 years of age and are presented in Table 3.

Four hundred of the 664 (60%) hysterectomies undertaken and 126 of the 204 (62%) done before 50 years of age were performed before cancer was diagnosed in any organ. Similarly, of the 598 women who had BSO, 328 (55%) had no prior or prevalent cancer at the time of the BSO, and among the 188 who had BSO before 50 years of age, 107 (57%) had no prior or prevalent cancer at the time of the BSO. Thus, the majority of the procedures were performed *as first major abdominal surgery* on young carriers without current or previous cancer. Among the 188 who underwent BSO before 50 years, the BSO was performed *after CRC as further abdominal surgery* in 64 (34%), and among these procedures, nine (4.8%) BSO and 11 (5.4%) hysterectomies were undertaken at the same age as CRC was diagnosed and 6 (3.2%) and 14 (6.9%) before the age of first CRC (Table 2). Thus, the majority of premenopausal RRS in women who had CRC were performed before first CRC, although in the cohort as a whole, the mean age at diagnosis of CRC was lower than the age at RRS.

## 4. Discussion

In this report, we provide information on the frequency and timing of risk-reducing hysterectomy and/or BSO by age and gene in female *path_MMR* carriers. The findings complement our previous reports on cumulative risks and mortality associated with gynaecological cancers in LS by age and gene [10,13]. We do not make management recommendations at this time, but our findings may inform future guidelines.

Although current guidelines recommend that hysterectomy and BSO are offered to *path_MMR* carriers to reduce their gynaecological cancer risk [14], PLSD data demonstrate that the uptake of RRS is only 26–36% in *path_MLH1*, *path_MSH2, and path_MSH6* and 19% in *path_PMS2* carriers. In the oldest cohort investigated in the present study, comprising 60- to 69-years-olds, 39–59% of *path_MLH1/MSH2* and *path_MSH6* carriers had undergone RRS. The reasons behind decisions made for or against RRS warrant further attention. For carriers of *path_PMS2*, the place for prophylactic surgery is still under debate because there is no good evidence of increased risk for ovarian cancer. Yet, 9–14% of *path_PMS2* carriers had undergone RRS.

We have recently published the estimates of the preventive impact of RRS. Risk-reducing hysterectomy at 25 years of age prevents endometrial cancer before 50 years in 15%, 18%, 13%, and 0% of *path_MLH1, path_MSH2, path_MSH6*, and *path_PMS2* carriers and death in 2%, 2%, 1%, and 0%, respectively [13]. Risk-reducing BSO at 25 years of age prevents ovarian cancer before 50 years in 6%, 11%, 2%, and 0% and death in 1%, 2%, 0%, and 0%, respectively. In line with the low risk for either endometrial or ovarian cancer before 40 years of age and the family planning considerations for this group, we found the uptake of hysterectomy was low before 40 years of age. Before 50 years of age, 21% of *path_MLH1* and *path_MSH2* carriers underwent hysterectomy compared with only 13% of *path_MSH6* carriers, despite the latter having similar cumulative risk for endometrial cancer. A difference in uptake was observed at older ages as well, but not to the same extent. The uptake of BSO was slightly lower and followed the same pattern, although *path_MSH6* carriers have a very low risk for ovarian cancer before 50 years of age. Notably, several *path_PMS2* carriers had premenopausal oophorectomy despite there being no evidence for increased risk for ovarian cancer either before or after the menopause [9,10], which is known to cause a negative impact on sexual health and endocrine symptoms [15].

Most surgical procedures were combined hysterectomy and BSO, irrespective of age, perhaps reflecting a desire to minimise gynaecological cancer risk ‘once and for all’. Modern-day minimally invasive surgical techniques may have fewer peri- and post-operative complications so that separate postmenopausal BSO may now be a reasonable option. Hysterectomy combined with BSO after 50 years of age for *path_PMS2* carriers effectively removes the gynaecological cancer risk. For younger carriers keen to mitigate their risks but also to avoid the surgical menopause, hysterectomy at the completion of childbearing followed by BSO at age 50 years would be an option for *path_MLH1, path_MSH2*, and particularly for *path_MSH6* carriers, in whom the risk of premenopausal ovarian cancer is low.

Because genetic testing has been available for only 25 years and identification of LS has been changing from phenotype/family history–based to molecular screening based, there may be a time-trend bias in the uptake of risk-reducing hysterectomy and BSO. Older women may not have had the option of early RRS that has been advocated and available in recent years (and they may not have known they were at risk when they were younger). The uptake we observed among older women may not be representative of the choices made by younger carriers today. Because of the inherent time-trend bias, from which no statistical procedures can escape, we considered it inappropriate to investigate the reported uptake of interventions using more sophisticated statistical methods than those selected for this study.

In addition to time trends, this study has other limitations. We have not recorded the exact indication for gynaecological organ removal, that is, whether this was risk reducing or conducted for benign medical indications, such as to manage menstrual dysfunction, fibroids, or benign ovarian masses. On some occasions, benign indications may favour earlier RRS than otherwise indicated. Some limitations are associated with the structure of PLSD that does not take into account whether the *path_MMR* variant in an individual had already been identified at the time of prospective observation, although it is now usually a prerequisite for recommending RRS. One may argue, however, that the increased incidence of endometrial and ovarian cancer in LS has been known throughout the observation period. In addition, the numbers of *path_PMS2* and *path_EPCAM* recorded in PLSD are still low, reflecting the insensitivity of the Amsterdam and Bethesda criteria so that they are infrequently offered genetic testing [16] and causing wide confidence intervals, particularly for younger cohorts.

This report and others from the PLSD, including reports on the guidelines that contributing centres have been following historically [7], their current guidelines [17], the reduction in morbidity and mortality achieved via hysterectomy or BSO by age [13], and now the uptake of hysterectomy or BSO by age and gene provide information that should help stakeholders, including patients, to address questions surrounding management options. Some patients may prefer to minimise the number of surgical procedures, some may wish to avoid the surgically induced menopause, and some may wish to maximise the cancer prevention effect of prophylactic organ removal [18]. Our results show that premenopausal women who had CRC most often had RRS performed as subsequent abdominal surgery, which increases risks for intraoperative complications and long-term complications such as hernias [19]. Although a staged approach will retain ovarian function for additional time, hormone replacement therapy is generally not contraindicated for women with LS, and adding simultaneous RRS to surgery for CRC in known *path_MMR* has been shown to be cost-effective and improve cancer outcomes in a Markov decision-tree model [20].

In summary, we found that uptake of RRS in LS aligned poorly with gynaecological cancer risk and mortality, both before and after menopause, with the timing of other abdominal surgery and with respect to clinical guidelines. Timing of RRS would benefit from earlier identification of LS, and there appears to be an unmet need for better multidisciplinary planning of prophylactic procedures to avoid repeated surgery. Today, the healthy young relatives of *path_MMR* carriers are increasingly being identified through genetic testing, and there is a need for timely presentation of options to these patients based on high-quality evidence.

## Supplementary Material

Suppl Materials

## Figures and Tables

**Table 1:** Mean age at the end of observation, at first risk-reducing gynaecological surgery (RRS) and at first colorectal cancer (CRC) diagnosis, by gene.

		Mean	SD	±95% CI
Age at last observation				
n = 1016	*path_MLH1*	48.7	10.2	0.6
n = 833	*path_MSH2*	48.7	10.3	0.7
n = 271	*path_MSH6*	49.9	10.3	1.2
n = 152	*path_PMS2*	54.0	10.3	1.6
n = 20	*path_EPCAM*	50.0	14.3	6.3
Age at first RRS				
n = 342	*path_MLH1*	45.4	7.6	0.8
n = 299	*path_MSH2*	44.4	7.9	0.9
n = 70	*path_MSH6*	47.6	8.3	1.9
n = 29	*path_PMS2*	48.3	9.8	3.6
n = 3	*path_EPCAM*	53.3	11.0	12.4
Age at first CRC				
n = 388	*path_MLH1*	40.8	9.1	0.9
n = 283	*path_MSH2*	40.8	9.7	1.1
n = 52	*path_MSH6*	43.7	8.4	2.3
n = 38	*path_PMS2*	46.8	8.3	2.6
n = 8	*path_EPCAM*	45.4	14.8	10.3

SD, standard deviation; CI, confidence interval (for mean point estimate); CRC, colorectal cancer.

**Table 2:** Numbers of risk-reducing gynaecological surgery (RRS) events with respect to previous or future cancers (percentage of all who underwent RRS^a^).

		All (30–69 years)	RRS at 30–49 years
All			
	Hysterectomy	664	204
	BSO	598	188
No prior or prevalent cancer			
	Hysterectomy	400 (60%)	126 (62%)
	BSO	328 (55%)	107 (57%)
CRC at same age as RRS			
	Hysterectomy	50 (7.5%)	11 (5.4%)
	BSO	41 (6.9%)	9 (4.8%)
CRC before RRS			
	Hysterectomy	197 (30%)	58 (28%)
	BSO	203 (34%)	64 (34%)
CRC after RRS			
	Hysterectomy	123 (19%)	14 (6.9%)
	BSO	92 (15%)	6 (3.2%)

BSO, bilateral salpingo-oophorectomy.

a Percentages do not sum to 100%, as some individuals are included in multiple groups.

**Table 3:** Cumulative uptake of risk-reducing hysterectomy with or without BSO or BSO with or without hysterectomy (±95% confidence interval) by gene and age. The table gives the figures corresponding to the graphical presentation in Fig. 1.

	Pathogenic variant	30–39 years				40–49 years				50–59 years				60–69 years				30–69 years			
		Number with or without RRS	Number with RRS	Frequency RRS	±95% CI	Number with or without RRS	Number with RRS	Frequency RRS	±95% CI	Number with or without RRS	Number with RRS	Frequency RRS	±95% CI	Number with or without RRS	Number with RRS	Frequency RRS	±95% CI	Number with or without RRS	Number with RRS	Sum carriers	Sum carriers with RRS
Hysterectomy and oophorectomy	*path_MLH1*	221	10	0.05	0.03	320	70	0.22	0.05	298	105	0.35	0.05	177	68	0.38	0.07	1016	263	2272	517
	*path_MSH2*	182	10	0.05	0.03	269	53	0.20	0.05	221	74	0.33	0.06	161	63	0.39	0.08	833	214		
	*path_MSH6*	53	1	0.02	0.04	73	8	0.11	0.07	91	23	0.25	0.09	54	17	0.31	0.12	271	51		
	*path_PMS2*	18	2	0.11	0.15	32	3	0.09	0.10	49	5	0.10	0.08	53	5	0.09	0.08	152	17		
Oophorectomy without hysterectomy	*path_MLH1*	221	0	0.00	0.00	320	12	0.04	0.02	298	10	0.03	0.02	177	7	0.04	0.03	1016	29	2272	79
	*path_MSH2*	182	2	0.01	0.02	269	13	0.05	0.03	221	14	0.06	0.03	161	11	0.07	0.04	833	40		
	*path_MSH6*	53	1	0.02	0.04	73	2	0.03	0.04	91	2	0.02	0.03	54	2	0.04	0.05	271	7		
	*path_PMS2*	18	0	0.00	0.00	32	1	0.03	0.06	49	2	0.04	0.06	53	0	0.00	0.00	152	3		
Hysterectomy without oophorectomy	*path_MLH1*	221	4	0.02	0.02	320	20	0.06	0.03	298	22	0.07	0.03	177	14	0.08	0.04	1016	60	2272	144
	*path_MSH2*	182	4	0.02	0.02	269	14	0.05	0.03	221	20	0.09	0.04	161	21	0.13	0.05	833	59		
	*path_MSH6*	53	2	0.04	0.05	73	3	0.04	0.05	91	7	0.08	0.05	54	2	0.04	0.05	271	14		
	*path_PMS2*	18	0	0.00	0.00	32	0	0,00	0,00	49	2	0.04	0.06	53	9	0.17	0.10	152	11		
Hysterectomy and/or oophorectomy	*path_MLH1*	221	14	0.06	0.03	320	102	0.32	0.05	298	137	0.46	0.06	177	89	0.50	0.07	1016	342	2272	740
	*path_MSH2*	182	16	0.09	0.04	269	80	0.30	0.05	221	108	0.49	0.07	161	95	0.59	0.08	833	299		
	*path_MSH6*	53	4	0.08	0.07	73	13	0.18	0.09	91	32	0.35	0.10	54	21	0.39	0.13	271	70		
	*path_PMS2*	18	2	0.11	0.15	32	4	0.13	0.11	49	9	0.18	0.11	53	14	0.26	0.12	152	29		
Hysterectomy	*path_MLH1*	221	14	0.06	0.03	320	90	0.28	0.05	298	127	0.43	0.06	177	82	0.46	0.07	1016	313	2272	661
	*path_MSH2*	182	14	0.08	0.04	269	67	0.25	0.05	221	94	0.43	0.07	161	84	0.52	0.08	833	259		
	*path_MSH6*	53	3	0.06	0.06	73	11	0.15	0.08	91	30	0.33	0.10	54	19	0.35	0.13	271	63		
	*path_PMS2*	18	2	0.11	0.15	32	3	0.09	0.10	49	7	0.14	0.10	53	14	0.26	0.12	152	26		
Oophorectomy	*path_MLH1*	221	10	0.05	0.03	320	82	0.26	0.05	298	115	0.39	0.06	177	75	0.42	0.07	1016	282	2272	596
	*path_MSH2*	182	12	0.07	0.04	269	66	0.25	0.05	221	88	0.40	0.06	161	74	0.46	0.08	833	240		
	*path_MSH6*	53	2	0.04	0.05	73	10	0.14	0.08	91	25	0.27	0.09	54	19	0.35	0.13	271	56		
	*path_PMS2*	18	2	0.11	0.15	32	4	0.13	0.11	49	7	0.14	0.10	53	5	0.09	0.08	152	18		

RRS, risk-reducing gynaecological surgery; CI, confidence interval (for mean point estimate); BSO, bilateral salpingo-oophorectomy.
