# Supplementary material for: Uptake of hysterectomy and bilateral salpingo-oophorectomy in carriers of pathogenic mismatch repair variants: a Prospective Lynch Syndrome Database report
Source: Eur J Cancer. Author manuscript; Available in PMC 2022 Mar 11. (PMC8916840; doi:10.1016/j.ejca.2021.02.022)
Supplement: Suppl Materials [file NIHMS1781077-supplement-Suppl_Materials.docx]

**Supplementary table 1.**

| Country | n | Mean age at last observation | SD | ± 95% CI |
| --- | --- | --- | --- | --- |
| AUSTRALIA | 331 | 50.5 | 11 | 1.2 |
| GERMANY | 326 | 48.1 | 9.8 | 1.1 |
| FINLAND | 304 | 49.6 | 10.8 | 1.2 |
| DENMARK | 292 | 48.2 | 10.2 | 1.2 |
| UK | 218 | 50.1 | 10.4 | 1.4 |
| SPAIN | 216 | 46.8 | 9.7 | 1.3 |
| USA | 126 | 52 | 9.2 | 1.6 |
| HOLLAND | 98 | 53.1 | 10.7 | 2.1 |
| NORWAY | 97 | 46.9 | 9.8 | 2 |
| SWEDEN | 69 | 49.5 | 10.4 | 2.5 |
| ITALY | 67 | 45.1 | 9.1 | 2.2 |
| CANADA | 57 | 50.4 | 10.1 | 2.6 |
| NEW_ZEALAND | 29 | 50.4 | 12.5 | 4.6 |
| URUGUAY | 24 | 49.2 | 10.9 | 4.4 |
| CHILE | 15 | 50.6 | 9.7 | 4.9 |
| ISRAEL | 10 | 45.6 | 8.1 | 5 |
| SWITZERLAND | 8 | 55 | 10 | 6.9 |
| ARGENTINA | 5 | 42.8 | 5.8 | 5.1 |
| Sum | 2292 |  |  |  |

SD, standard deviation

CI, confidence interval (for mean point estimate)
